# Evaluation of microRNA expression in a sheep model for lung fibrosis

**DOI:** 10.1186/s12864-021-08073-4

**Published:** 2021-11-17

**Authors:** Udari Eshani Perera, Habtamu B. Derseh, Sasika N. V. Dewage, Andrew Stent, Rukmali Wijayarathna, Kenneth J. Snibson

**Affiliations:** 1 School of Veterinary Science, The University of Melbourne, Parkville, VIC Australia; 2 Walter and Eliza Hall Institute of Medical Research, Parkville, Australia; 3 School of Veterinary Science, The University of Melbourne, Werribee, VIC Australia; 4 Centre for Reproductive Health, Hudson Institute of Medical Research, Australia and Monash University, Clayton, VIC Australia

**Keywords:** Sheep model, microRNA, Idiopathic pulmonary fibrosis, Bleomycin

## Abstract

**Background:**

Idiopathic pulmonary fibrosis (IPF) is a chronic progressive fibroproliferative disorder that has one of the poorest prognoses amongst interstitial lung diseases. Recently, the finding of aberrant expression levels of miRNAs in IPF patients has drawn significant attention to the involvement of these molecules in the pathogenesis of this disease. Clarification of the differential expression of miRNAs in health and disease may identify novel therapeutic strategies that can be employed in the future to combat IPF. This study evaluates the miRNA expression profiles in a sheep model for lung fibrosis and compares them to the miRNA profiles of both IPF patients and the mouse bleomycin model for pulmonary fibrosis. Pathway enrichment analyses were performed on differentially expressed miRNAs to illustrate which biological mechanisms were associated with lung fibrosis.

**Results:**

We discovered 49 differentially expressed miRNAs in the sheep fibrosis model, in which 32 miRNAs were significantly down regulated, while 17 miRNAs were significantly upregulated due to bleomycin-induced lung injury. Moreover, the miRNA families miR-29, miR-26, miR-30, let-7, miR-21, miR-19, miR-17 and miR-199 were aberrantly expressed in both sheep and mouse models, with similar differential miRNAs expression observed in IPF cases. Importantly, 18 miRNAs were aberrantly expressed in both the sheep model and IPF patients, but not in mice.

**Conclusion:**

Together with pathway enrichment analyses, these results show that the sheep model can potentially be used to characterize previously unrecognized biological pathways associated with lung fibrosis.

## Background

Idiopathic pulmonary fibrosis (IPF) is a chronic progressive fibroproliferative disorder categorized under the umbrella of interstitial lung diseases [1–3]. Despite significant progress in understanding the pathogenic mechanisms of the disease, the molecular pathogenesis of the disease remains unclear [3]. MicroRNAs (miRNAs) are known to play critical roles in modifying a wide range of biological processes, including tissue development and differentiation; cellular proliferation; tissue repair; and regulation of intracellular cell signaling pathways. Many of these processes are frequent changes that occur in a variety of diseases, including IPF [2, 4, 5].

miRNAs are phylogenetically conserved small non-coding short RNA sequences, 19–22 nucleotides in length, that are generated by a Dicer enzyme from hairpin-shaped single-stranded RNA precursors composed of about 70–90 base pairs [2, 3, 6, 7]. Each miRNA has several targets and several microRNA can also regulate the same gene [2]. The main function of microRNA is to inhibit protein synthesis either by inhibiting the translation, or degradation, of mRNA [1, 5, 8].

Recently, miRNA has drawn significant attention due to its aberrant expression in human IPF. Studies published over the last 10 years report that approximately 10% of miRNAs are altered in an IPF setting [4, 9]. However, the investigation of miRNAs in IPF is still at an early stage [3]. Even though microRNA expression profiles have been reported, miRNA-mRNA interactions and pathway enrichment related to IPF have yet to be comprehensively explored [3]. Increased understanding of miRNA expression profiles and their influence on biological process in IPF is therefore important for identification of potential biomarkers and therapeutic targets for IPF [10–12].

Animal models have been used in the preclinical setting to study the pathogenesis in IPF. We have developed a novel sheep model for lung fibrosis using bleomycin, which shares many of the characteristic features of IPF [13]. The model has been used to test and compare candidate anti-fibrotic drugs [14–16] to the FDA-approved drug pirfenidone (which is efficacious in the sheep model [17]. This is the first report evaluating the expression profiles of miRNAs under experimental conditions using the sheep model for lung fibrosis. After defining the miRNA expression profiles in the sheep model for lung fibrosis, these profiles were compared with those reported in the literature for IPF patients and in mouse lung fibrosis models. We also performed a pathway enrichment programs on the differentially expressed miRNA to illustrate the various biological mechanisms associated with lung fibrosis.

## Results

### Segmental lung compliance reduced when the lung fibrosis increases at week 4

**Fig. 1:**
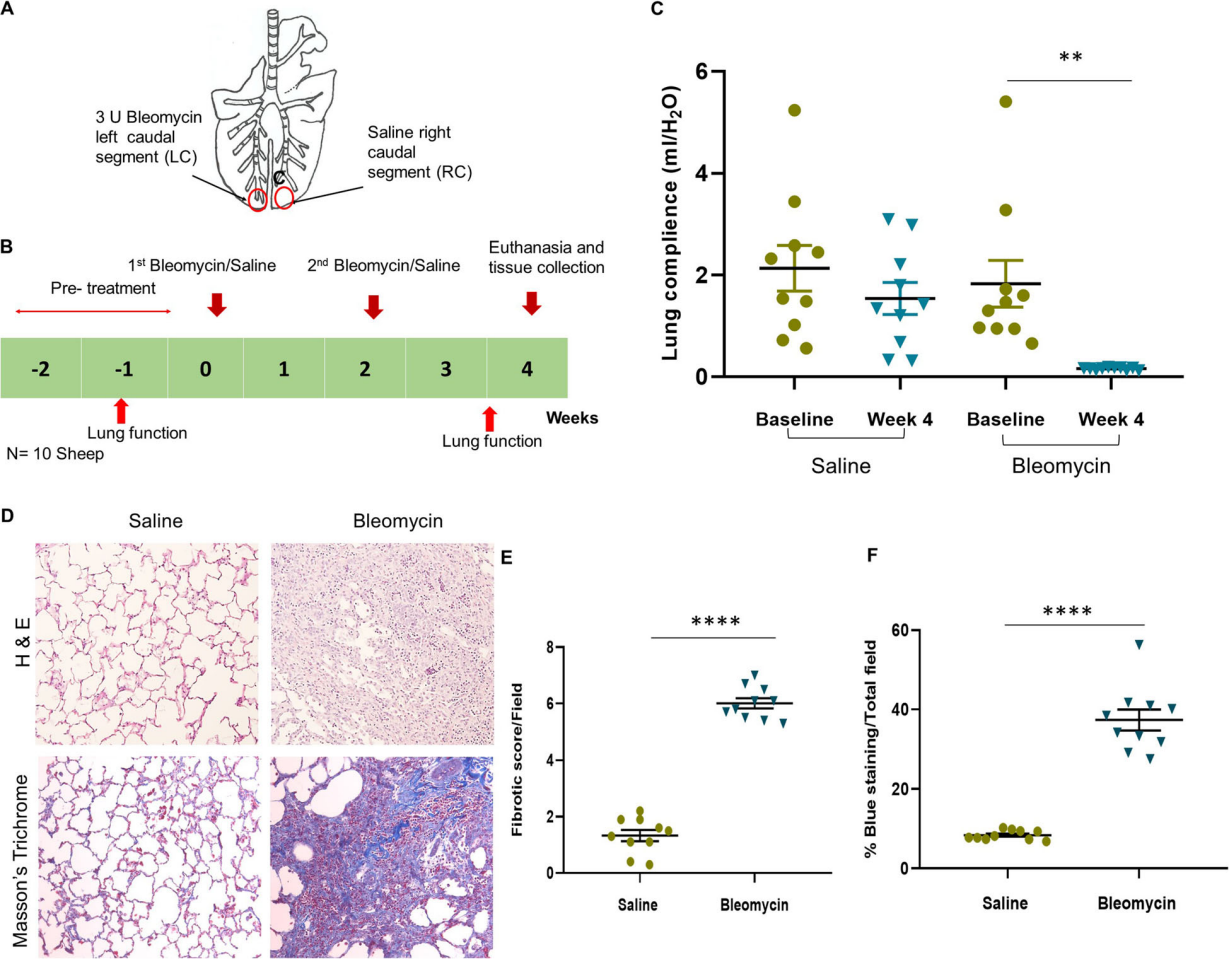
Bleomycin administration protocol, lung compliance and histological evaluation of lung fibrosis. **A** In individual sheep (n=10), the left caudal lung segment received infusions of bleomycin, while the right caudal lung segment received the saline for controls. **B** Schematic diagram of bleomycin infusion protocols in sheep model. **C** Segmental lung compliance at the baseline and week 4. **D)** H & E staining was used to evaluate fibrotic score, Masson’s trichrome staining was used to evaluate % of blue staining. Ten representative randomly selected areas from each lung tissue sections, were captured under ×20 magnification for the analysis. **E** Fibrotic scores were expressed as median and interquartile range and **(F)** fibrotic fraction were expressed as means and standard errors of means. Significance was determined using Student’s t- test and Mann Whitney test denoted as follows, ** *p*< 0.005, **** *p*< 0.0001

Lung function was assessed by measuring the segmental compliance (Cseg) in saline- and bleomycin-infused lung lobes at baseline and week 4 (Fig. 1C). The mean segmental compliance was significantly reduced following the bleomycin infusion in lung segments at week 4. In contrast, the lung compliance of the saline-infused control lung segments remained the same at the baseline and week 4.

Our findings were further supported by the histopathology of lung segments stained with H & E and Masson’s trichrome stain. Moderate to severe irregular, multifocal collagen deposits were observed in the bleomycin-infused sheep lung segments. These excessive collagen and connective tissue depositions resulted in irregular thickening of alveolar septa. In contrast, the saline infused lung segments displayed minimal fibrotic changes and were consistent with healthy lung tissues. The highest fibrosis score was observed in the lung segments infused with bleomycin when compared to saline lung segments (Fig. 1E and F). Furthermore, an assessment of Masson’s trichrome-stained lung tissues showed that the percentage of blue (a measure of connective tissue content) was significantly increased in bleomycin-infused lung segments compared to saline control segments (Fig. 1D and F).

### Differentially expressed miRNA profile in sheep model for lung fibrosis

**Fig. 2:**
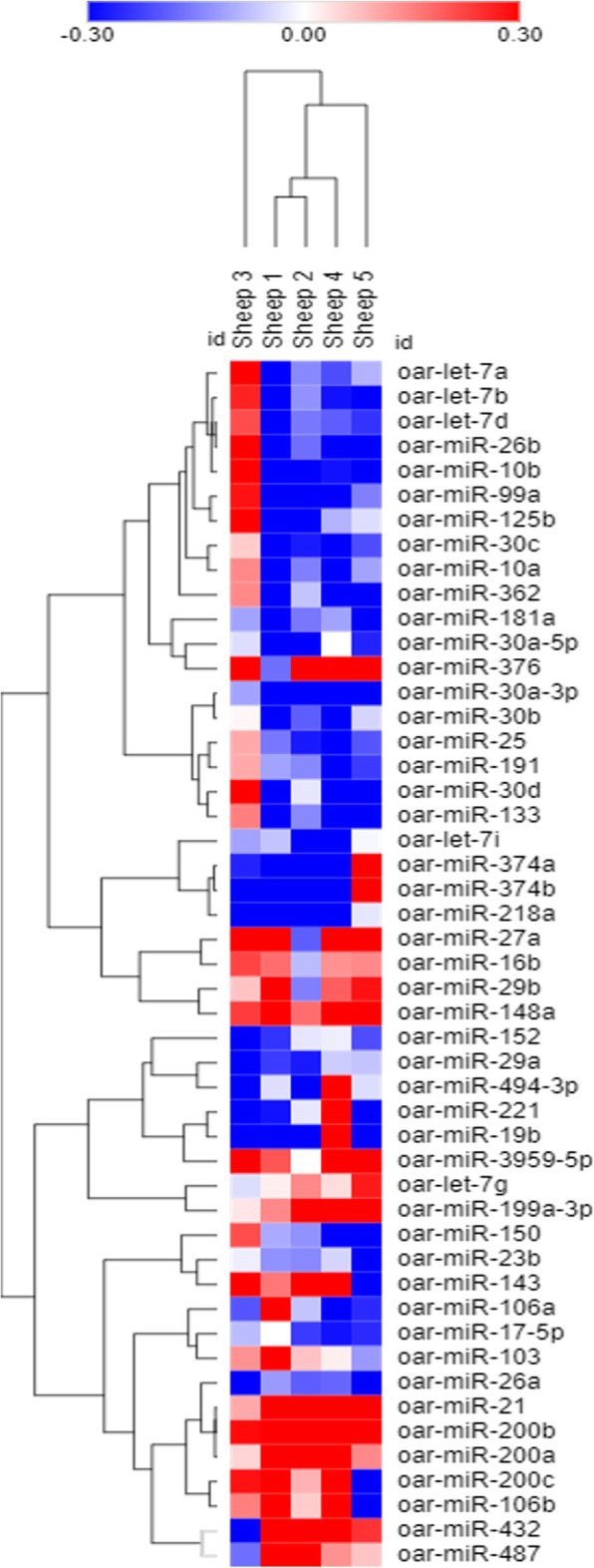
Heatmap of significantly expressed miRNA microarray expression profile in the sheep model for lung fibrosis. The expression level of each miR was presented as fold-change relative to the control/saline group. Average intensity from the 21 replicates were taken for each miRNA probes tested for bleomycin and saline/control lung segments. Student’s t-test was performed and *p* < 0.01 was considered statistically significant. Hierarchical clustering was performed by applying One minus Pearson correlation to cluster differentially expressed miRNAs. Blue indicates under expression and red indicates over expression

miRNA profiles were evaluated in 5 sheep, which were selected based on the low lung compliance and high fibrotic scores. The lung tissues were obtained to evaluate the changes in the miRNA expression levels at week 4 when the bleomycin injury is optimal. The data taken from 5 sheep showed that 49 miRNAs were differentially expressed due to bleomycin lung injury (Fig. 2). Out of those, 32 miRNAs were significantly downregulated in bleomycin-infused lung segments compared to the control/saline infused lung segments. In addition, 17 miRNAs were significantly up regulated in bleomycin-infused lung segments at week 4 when compared to saline infused lung segments (Fig. 2) (Table 1).

**Table 1:** Significantly expressed microRNA microarray profile in sheep model for lung fibrosis

Downregulated miRNA	Upregulated miRNA
oar-miR-181a oar-miR-23b oar-let-7a oar-let-7b oar-let-7d oar-let-7i oar-miR-25 oar-miR-26a oar-miR-26b oar-miR-29a oar-miR-30a-5p oar-miR-30a-3p oar-miR-30b oar-miR-30c oar-miR-30d oar-miR-10a oar-miR-10b oar-miR-99a oar-miR-150 oar-miR-152 oar-miR-374a oar-miR-374b oar-miR-133 oar-miR-218a oar-miR-125b oar-miR-191 oar-miR-106a oar-miR-221 oar-miR-362 oar-miR-494-3p oar-miR-17-5p oar-miR-19b	oar-miR-21 oar-miR-199a-3p oar-miR-103 oar-miR-29b oar-let-7 g oar-miR-27a oar-miR-200a oar-miR-200b oar-miR-200c oar-miR-432 oar-miR-16b oar-miR-106b oar-miR-148a oar-miR-376 oar-miR-143 oar-miR-487 oar-miR-3959-5p

**Fig. 3:**
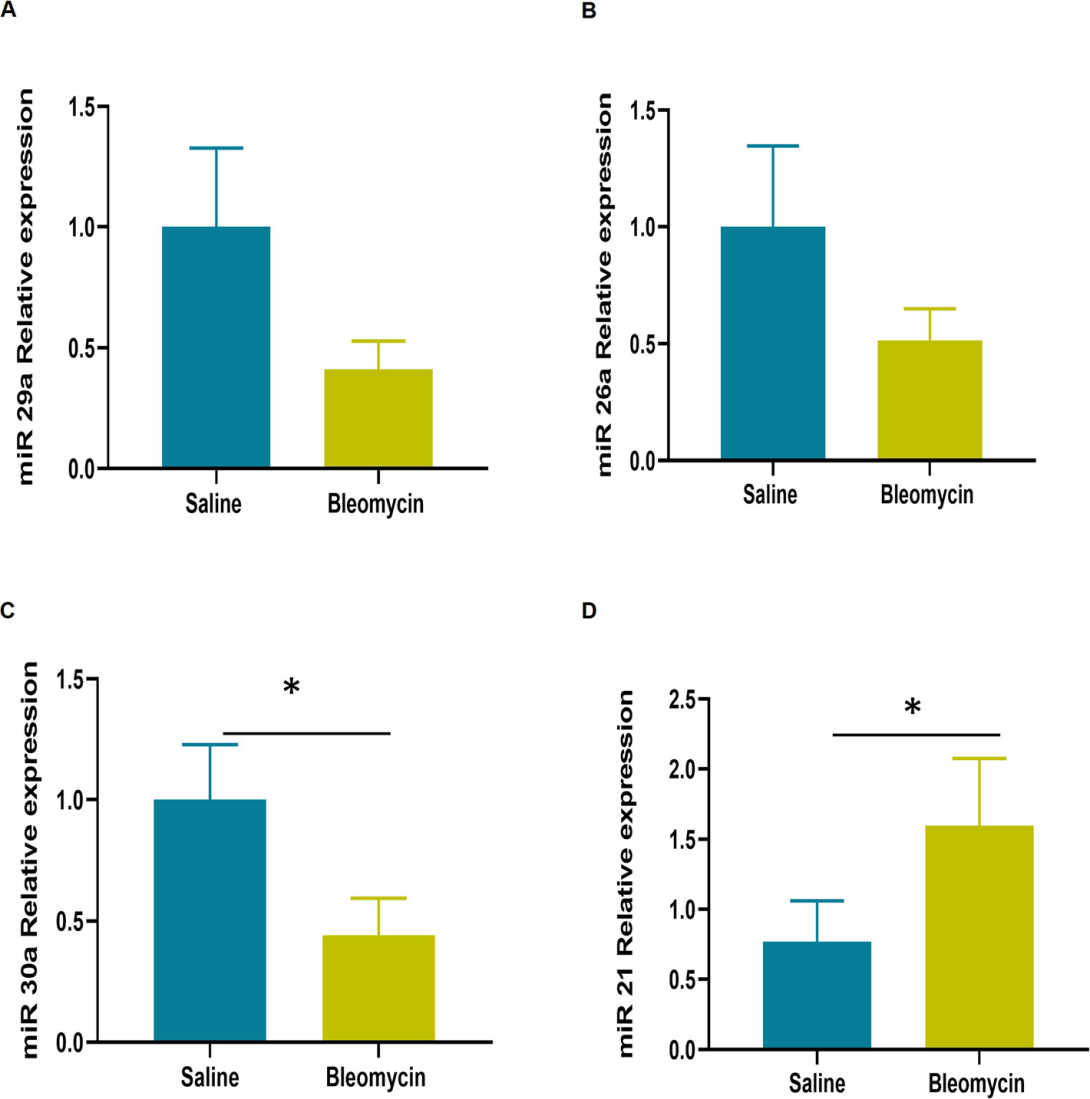
Real-time PCR relative expression of miR-29a, miR-26a, miR-30a and miR-21 in sheep lung segments infused with bleomycin (*n* = 5) and saline (n = 5). Significance was determined using student’s t- test and denoted as follows, * *p* < 0.05

Four miRNAs - miR-29a, miR-26a, miR-30a and miR-21 - that were differentially expressed in the miRNA microarray were examined by qPCR for validation. Bleomycin injury was associated with a significant downregulation of miR-30a, while miR-29a and miR-26a showed a similar but non-significant downregulation trend with bleomycin infusion (Fig. 3A, B & C). Furthermore, bleomycin induced lung damage was associated with a significant upregulation of miR-21 expression (Fig. 3D) in sheep. Overall, for each of these miRNAs, the up- or downregulation expression pattern was similar in both the microarray and the qPCR assays.

**Fig. 4:**
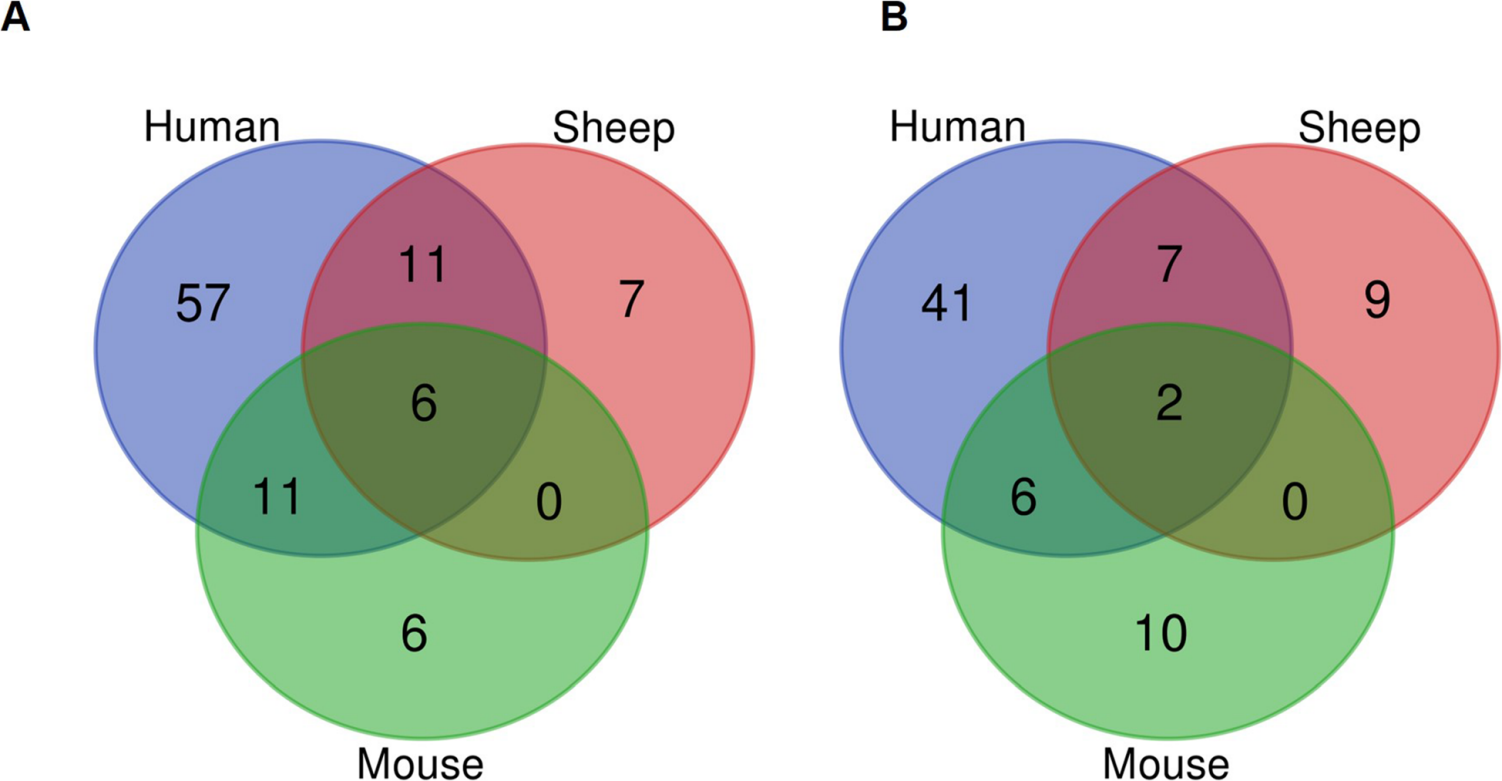
Venn diagrams of differentially expressed microRNA in IPF patients and sheep and mouse models for lung fibrosis reported between the period of 2010–2020. Data were analysed using the Bioinformatic and Evolutionary Genomics software **A)** Down-regulated microRNA. **B)** Up-regulated microRNA

Comparative evaluation of miRNA profiles showed that 6 miRNA families (miR-26, miR-29, miR-30, let-7, miR-17, miR-19) were downregulated in lung fibrosis for all three species; human, sheep, and mouse (Table 2 and Fig. 4A). In addition, 11 miRNA families were downregulated similarly in IPF patients and sheep models, but not in mice, while a different group of 11 miRNA families were downregulated in IPF patients and bleomycin damaged mouse lungs, but not in fibrotic sheep lungs (Table 2 and Fig. 4A).

**Table 2:** Comparative analysis of microRNA in IPF patients and sheep and mouse models for lung fibrosis

Dysfunction	miRNAs in human, sheep, and mice	miRNAs in human and sheep	miRNAs in human and mouse	Reference
	let-7	miR-181	miR-27	[1, 2, 6, 8, 12, 18–33]
	miR-29	miR-23	miR-130	
	miR-30	miR-25	miR-326	
Downregulated	miR-26	miR-99	miR-18	
	miR-19	miR-150	miR-20	
	miR-17	miR-374	miR-92	
		miR-125	miR-200	
		miR-191	miR-157	
		miR-106	miR-140	
		miR-221	miR-145	
		miR-362	miR-338	
	miR-21	miR-432	miR-142	[1, 2, 6, 8, 18, 19, 23, 25, 26, 28, 31, 33–35]
	miR-199	miR-376	miR-155	
		miR-487	miR-449	
Upregulated		miR-200b	miR-133	
		miR-200c	miR-96	
		miR-27	miR-34	
		miR-143

Two miRNA families (miR-21 and miR-199) were upregulated in the two animal species (sheep and mouse), as well as in IPF patients, (Fig. 4B, Table 2). Furthermore, we identified 7 miRNA families where upregulation was restricted to IPF patients and sheep lung fibrosis (not mice), and a different set of 6 miRNA families where the upregulation was restricted to IPF patients and mice (not sheep) (Table 2 and Fig. 4B).

Overall, the sheep model and IPF patients had a total of 18 miRNAs in common that were differentially expressed, while the mouse model and IPF patients had a total of 17 miRNAs in common that were differentially expressed. Furthermore, around 98 miRNAs (Fig. 4) that were detected in IPF patients were not detected in the sheep and mouse models of lung fibrosis.

### GO analysis and KEGG pathway analyses

**Fig. 5:**
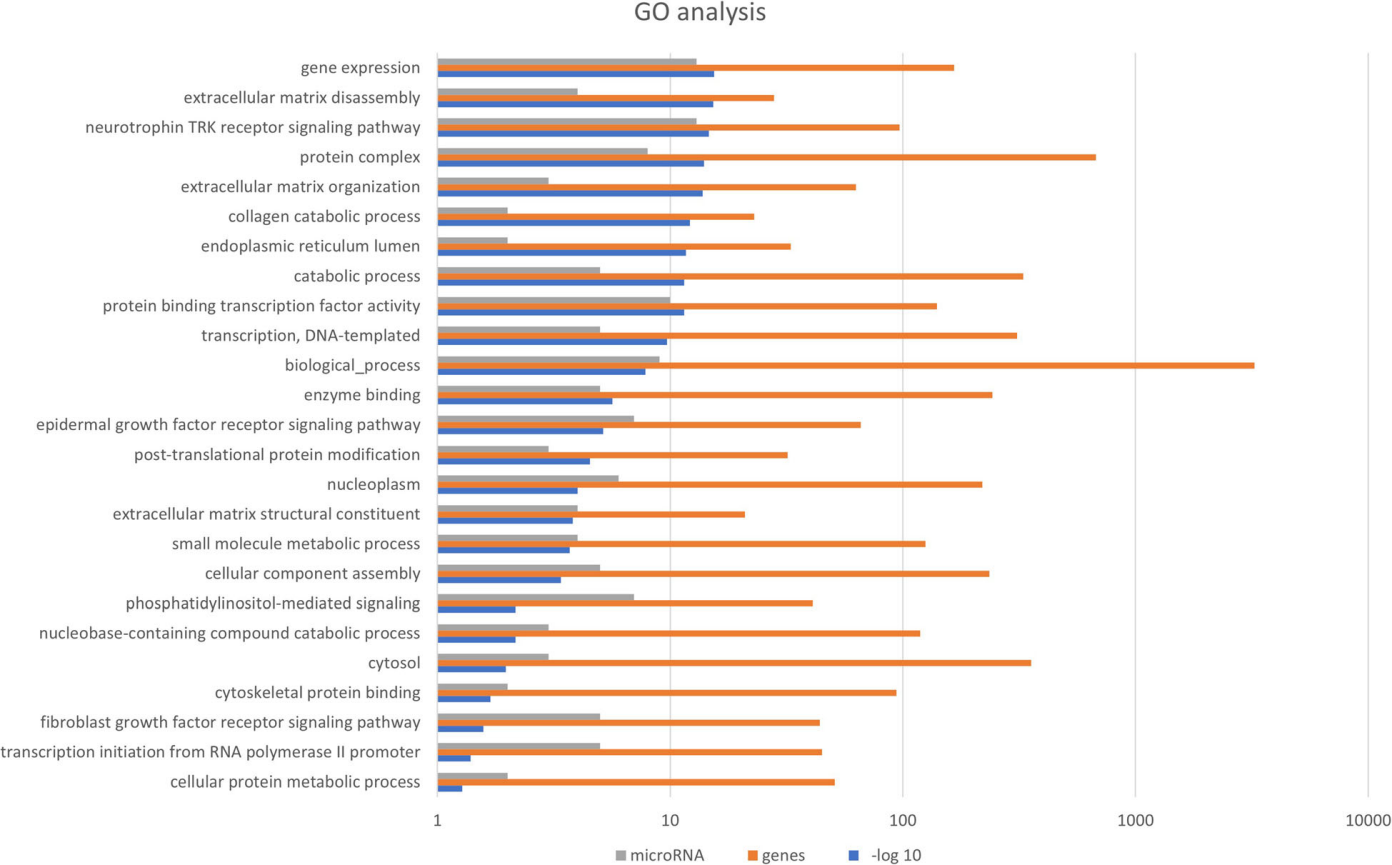
GO enrichment analysis (biological process, cellular component, and molecular function) of differentially expressed microRNA in sheep model of lung fibrosis. Go analysis was performed using DIANA mirPath v.3 software. microT- CDS and TargetScan were utilized to identify microRNA- gene interactions. Significance was determined by performing Fisher’s Exact Test. *p* value of *p* < 0.05 was considered statistically significant

Pathway enrichment analyses were performed on 49 differentially expressed miRNAs in the sheep model for lung fibrosis using DIANA mirPath v.3 software. A total of 25 signaling pathways were identified as the most significantly enriched pathways according to their p- value (*p* < 0.05). According to the GO analysis, the differentially expressed miRNAs were mainly enriched for GO terms related to gene expression, extracellular matrix disassembly, neurotrophin TRK receptor signaling pathway, extracellular matrix organization, collagen catabolic process, endoplasmic reticulum lumen, extracellular matrix structural constituent and fibroblast growth factor receptor signaling pathways (Fig. 5).

**Fig. 6:**
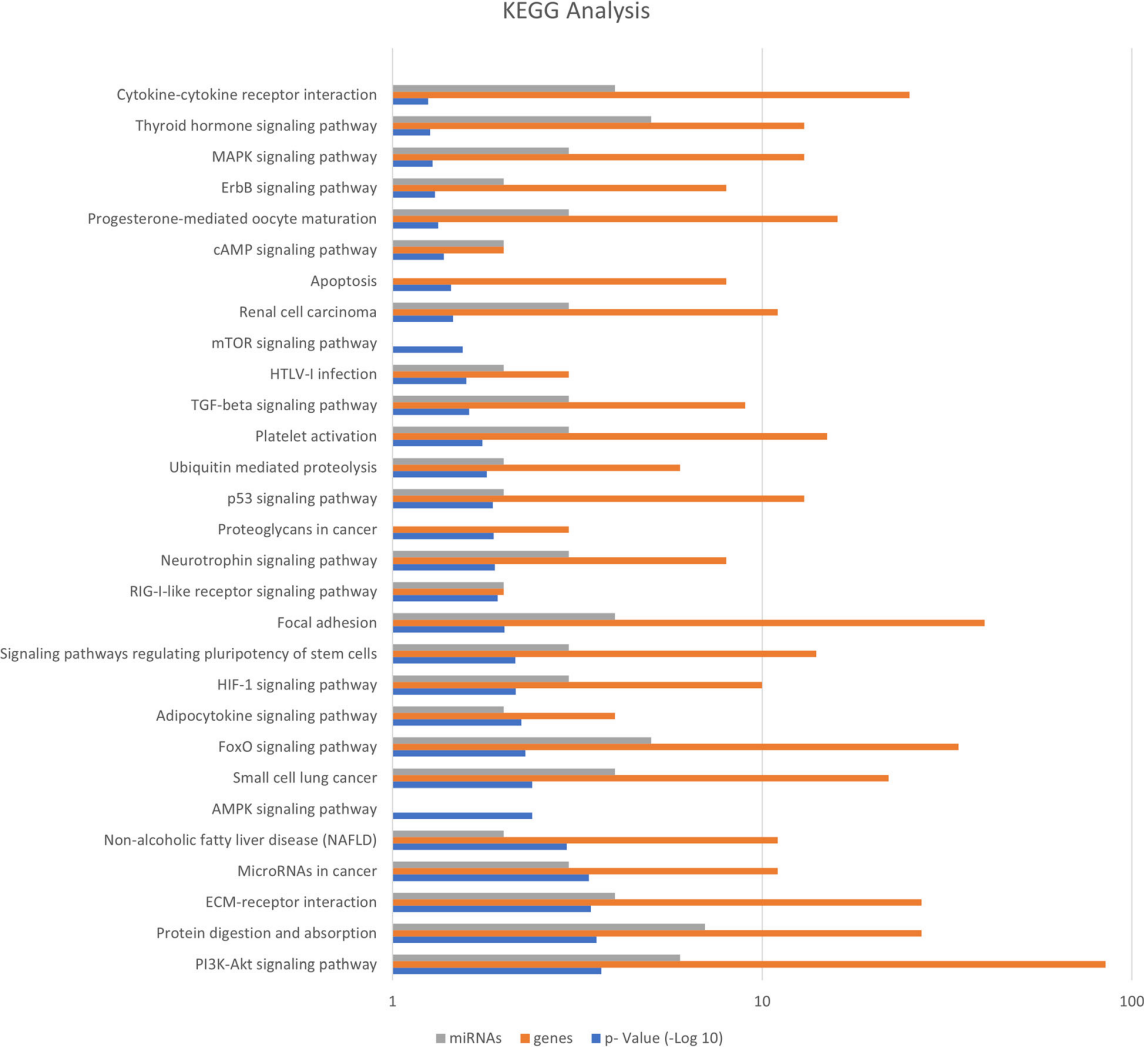
KEGG pathway analysis of differentially expressed microRNA in sheep model of lung fibrosis. KEGG pathway analysis was performed using DIANA mirPath v.3 software. microT- CDS and TargetScan were utilized to identify microRNA- gene interactions. Significance was assessed by performing Fisher’s Exact Test. p value of p < 0.05 was considered statistically significant

KEGG pathway analysis was used to determine the biological pathways associated with the differentially expressed miRNAs in sheep model for lung fibrosis. KEGG pathway analysis demonstrated that the dysregulated miRNA could be enriched significantly into 29 signalling pathways according to their *p*-value (*p* < 0.05) (Fig. 6). PI3K-Akt signalling pathway, protein digestion and absorption, extracellular matrix receptor interaction, TGF-β signalling pathway, miRNAs in cancer, small cell lung cancer, neurotrophin signalling pathway, p53 signalling pathway, platelet activation, apoptosis, and ErbB signalling pathway are some of the predominant pathways. We performed a comparative evaluation of biological pathways enriched by differential expression of microRNAs in IPF patients and sheep and mice (Table 3).

**Table 3:** Comparative evaluation of biological pathways enriched by differential expression of microRNA

Biological pathways enriched in IPF patients	Sheep model	Mouse model
[11, 31, 36]		[26, 37]
TGF-beta signalling pathway	✓	✓
MAPK signalling pathway	✓	✓
PI3K–Akt signalling pathway	✓	
Insulin signalling pathway		
ErbB signalling pathway	✓	
Focal adhesion	✓	✓
Adherens junction	✓	✓
Wnt signalling pathway		✓
RNA degradation		
Ubiquitin mediated proteolysis	✓	
Endocytosis		
HIF-1 signalling pathway	✓	
Neurotrophin signalling pathway	✓	✓
Adipocytokine signalling pathway	✓	
Regulation of actin cytoskeleton		
Osteoclast differentiation		
Hepatitis B		✓
Jak–STAT signalling pathway		✓
mTOR signalling pathway	✓	✓
Notch signalling pathway	✓	
HTLV-I infection	✓	
Hedgehog signalling pathway		✓
Amoebiasis		
T cell receptor signalling pathway		
MicroRNAs in cancer	✓	✓
Cell cycle		✓
Proteoglycans in cancer		
Pathways in cancer		✓
Protein processing in endoplasmic reticulum	✓	
Renal cell carcinoma	✓	✓
Fatty acid metabolism		
Hippo signalling pathway		✓
Nuerotrophin TRK receptor signalling pathway	✓	
Epidermal growth factor receptor signalling pathway	✓	
Fibroblast growth factor receptor signalling pathway	✓	
ECM-receptor interaction	✓	
p53 signalling pathway	✓	
Gene expression	✓	
Apoptosis	✓	✓
Vascular smooth muscle contraction		
GnRH signalling pathway		
Pathways in cancer	✓	✓
VEGF signalling pathway	✓	
Calcium signalling pathway		

## Discussion

This study provides a comprehensive evaluation of the miRNA expression profile in a sheep model developed for lung fibrosis. We discovered a total of 49 differentially expressed miRNAs, 32 of which were significantly downregulated due to bleomycin induced lung injury, while 17 were significantly upregulated. Moreover, the differentially expressed miRNA families (miR-29, miR-26, miR-30, let-7, miR-21, miR-19, miR-17, miR-199) reported for IPF patients, were also differentially expressed in the bleomycin damaged lung tissues of both the sheep and mice [4, 19, 32, 38]. In addition, we found 18 miRNAs which were differentially expressed only in sheep and IPF fibrotic lungs, but not in bleomycin-injured mouse lungs. Pathway enrichment analyses of biological processes and signaling pathways of the 49 differentially expressed miRNAs, identified many pathways that were closely associated with IPF patients (Table 3) [11, 31, 36]. Moreover, these analyses revealed key miRNAs that play vital roles in important biological processes in fibrotic lungs. These processes include cell proliferation and apoptosis; cell movements; cellular metabolism; and intracellular signaling.

Amongst the 18 miRNAs that were differentially expressed in both the sheep model and IPF patients, but not in mice, 11 miRNAs; miR-181, miR-23, miR-25, miR-99, miR-150, miR-374, miR-125, miR-191, miR-221, miR-106 and miR-362 were similarly down-regulated and 7 miRNAs; miR-432, miR-487, miR-143, miR-376, miR-200b, miR-200c and miR-27 were up-regulated. Previous studies have investigated the mechanisms of some of these miRNAs. When miR-181 is transfection into Human Pulmonary Microvascular Endothelial cells (HPMEC) it was found to decrease Fas- induced apoptosis, while reducing inflammatory factors [39]. miR-23 has been found to be expressed in T cells and involved in the regulation of T cell function. Moreover, the aberrant expression of miR-23 resulted in the dysregulation of T cell activation and differentiation [40]. miR-374 can inhibit Fas-induced apoptosis in human primary retinal pigment epithelial (RPE) cells by targeting Fas during oxidative conditions. In particular, miR-374 overexpression restrained tumorigenicity and cell proliferation, while accelerating cell apoptosis through targeting Wnt-16 and AKT1, which inhibits AKT signal pathway [41]. miRNA-221 targets several genes that are involved in the TGF-β signaling, including JNK1 (c-Jun N-terminal kinase 1), TGF-β receptor 1 and TGF-β receptor 2, and ETS-1 (ETS proto-oncogene 1) [42]. Inhibition of miR-106 in Renal Cell Cancer cell lines altered the cell migration, invasion, and wound healing abilities [43]. miRNA miR-191 is expressed in T cells and acts as a key regulator of, memory, and regulatory T cell homeostasis [44]. While the above-mentioned studies give an insight into how these miRNAs might be operating, further investigations are needed to discover the contribution of these miRNAs to the pathogenesis of lung fibrosis, especially in larger animals.

While the alterations in the miRNA expression patterns have been discovered in IPF patients, the exact mechanisms of how these miRNA expressions change with the disease progression is still under investigation. Our findings suggest the molecular pathogenesis of the sheep bleomycin model for lung fibrosis shares some of the molecular mechanisms of IPF patients. This is an interesting finding due to the differences in age, duration of disease, etiology, and progressive nature of bleomycin induce lung fibrosis and IPF. Bleomycin induced lung fibrosis is an acute injury process with a known etiology, and apart from the recent exception [45], most animal models using bleomycin do not demonstrate the progressive nature of IPF [46]. In contrast, IPF is chronic and progressive disease condition with an unknown etiology. Despite these differences, both IPF and animal models of bleomycin-induced lung injury share common pathogenic mechanisms.

The miR-29, miR-26, miR-30, let-7, and miR-21 miRNA families were all differentially expressed in sheep, human and mice, and have been widely investigated as mediators in fibrotic disorders [5]. Previous studies showed that miR-29 is one of the main miRNAs involved in the pathogenesis of IPF [4, 47]. We showed that miR-29 is downregulated in the sheep model of lung fibrosis, similar to its expression patterns in IPF patients and fibrotic mouse lungs [48, 49]. Previous research showed that profibrotic factors such as transforming growth factor β (TGF- β), platelet derived growth factor (PDGF), interleukin-4 (IL-4) and tumor necrosis factor α (TNF-α) suppress miR-29 expression during the process of fibrosis [47]. Down regulation of miR-29 leads to overexpression of these profibrotic factors, causing further downregulation of miR-29 expression. This results in the promotion of collagen and extracellular matrix-related gene expression [5, 47]. Hence, miR-29 can directly regulate these vital processes that are important in the underlying mechanisms of pulmonary fibrosis [47].

miR-26a has been identified as potential therapeutic target that can be exploited to reduce fibrogenesis [18]. Expression of miR-26a was downregulated in bleomycin-damaged sheep and mouse lungs, as it is in IPF patients. Downregulation of miR-26a is likely via TGF-β1-induced activation of Smad3 [18]. Previous studies showed that miR-26a is involved in proliferation and differentiation of fibroblasts into myofibroblasts in IPF [12, 18]. Importantly, the overexpression of miR-26a attenuates bleomycin-induced pulmonary fibrosis in mice and TGF-β1-induced fibrogenesis in MRC-5 cells [18]. This suggests that it is a candidate for further investigation as a target to treat IPF [18].

Furthermore, we found that miR-30a expression was significantly downregulated due to bleomycin-induced lung injury in the sheep model. Our findings were in line with the low expression of miR-30a in IPF patients and mouse bleomycin models [38]. Previous studies showed that miR-30a was downregulated by 71.8% in the bronchoalveolar lavage fluids of IPF patients [38]. In the current study, KEGG analysis showed enrichment of TGF-β and MAPK signaling pathways, which is consistent with the findings of Liu [38] in which they showed that the miR-30a may directly interact with TGF-β activated kinase 1/MAP 3 K7 binding protein 3 (TAB3) mRNA. They also showed that overexpression of miR-30a attenuates TGF-β1-induced upregulation of TAB3, α-SMA and fibronectin expression in 293 T cells [38]. These studies suggest that miR-30a could be one of the key factors that supports progression of lung fibrosis in IPF patients.

Our study showed that miR-21 and miR-199 expression were upregulated in bleomycin-injured sheep and mouse lungs, consistent with findings in IPF lungs. High expression of miR-199 was observed in fibrotic foci of IPF lungs [50]. Furthermore, silencing miR-199a-5p strongly inhibited TGF-β1-mediated differentiation of fibroblasts into myofibroblasts, wound repair and SMAD signaling in MRC-5 cells [50]. The importance of upregulated miRNAs in the fibrotic processes has been shown in studies where downregulation of miR-21 results in the attenuation of bleomycin-induced lung fibrosis in mice [35]. miR-21 is induced by the TGF-β signaling pathway and amplifies TGF- β signaling in a positive feedback fashion [35]. In addition, miR-21 promotes epithelial mesenchymal transition and enhances miR-21expression in myofibroblasts [34, 35]. For these reasons, miR-21 is considered as a potential therapeutic target for IPF [35].

Bleomycin-infused sheep lung segments also showed downregulation in the members of let-7 family, consistent with findings in IPF patients and mouse bleomycin models [27, 32, 51]. Let-7 expression is mainly localized to the alveolar epithelium in normal lungs and a significant reduction was observed in alveolar epithelium due to inhibition by the key profibrotic cytokine TGF-β in IPF lungs [27]. Therefore, it has been identified as one of the key regulatory events responsible for the phenotypic changes occur in the alveolar epithelium of IPF lungs [27].

While this study used a microarray which profiles predefined transcripts to identify miRNAs involved in sheep lung fibrosis, future studies using whole transcriptome sequencing methods which capture the complete miRNA profile will enable the discovery of novel ovine miRNAs regulating fibrosis. Furthermore, mechanistic studies to delineate how the identified miRNAs regulate fibrotic genes are required.

Discovery of dysregulated miRNA expression profiles and the associated pathogenic mechanisms involved in the disease process of IPF potentially open new therapeutic avenues for IPF. In addition, miRNAs have also been considered as top potential biomarkers due to their upstream positions in regulation cascades which provide the benefit of an early diagnosis. While the alterations can be readily discovered by genomic tools such as microarrays and next generation sequencing, and even low level expression of miRNAs can be detected via qPCR which is much more accessible in a diagnostic setting [52].

## Conclusion

The novel insights of this study showed similarities in the miRNA expression profiles in a sheep model for lung fibrosis and IPF patients, while illustrating the useful pathogenic mechanisms associated with lung fibrosis, suggest that bleomycin induced lung injuries in the sheep model share some of the molecular mechanisms associated with human IPF. Importantly, some miRNAs were significantly expressed only in the sheep model and IPF patients. Hence, the sheep model can be potentially used as an additional tool to characterize the underlying molecular mechanisms associated with lung fibrosis.

## Materials and methods

### Experimental design

Healthy female merino sheep (*n* = 10) 9–12 months old were used as subjects for the pulmonary fibrosis model. The experimental procedures and tissue sample collections were approved by Animal Experimentation Ethics Committee, University of Melbourne (Parkville, VIC, Australia), which adheres to the ARRIVE guidelines **(**https://arriveguidelines.org**)** and the Australian Code of Practice for the care and use of laboratory animals for scientific purposes.

### Bleomycin administration protocol

Bleomycin was used to induce pulmonary fibrosis in the sheep. 5 ml of 3 U bleomycin/segment (0.6 U/ml bleomycin) and 5 ml of 0.9% sterile saline (control) were bronchoscopically administered to the left and the right caudal lung segments respectively, as described in our previous study [13] (Fig. 1A). The saline-infused lung segment serves as the internal control in the sheep model. Intra-bronchial bleomycin/saline were administered in two doses delivered 2 weeks apart to the appropriate lung segment via the biopsy port of the fiber-optic bronchoscope (Fig. 1B).

### Segmental lung function analysis

Wedged-bronchoscope technique was used to measure the changes in the segmental lung compliance which occur due to bleomycin at baseline and week 4 as described previously [13] (Fig. 1C).

### Necropsy and tissue sampling

Animals were euthanized at the end of week 4 by administering barbiturate (Lethabarb). Targeted lung segments were isolated during the necropsy and cannulated by exposing the bronchiole to inflate the segments. Next, a mixture of 1:1 optimal cutting temperature (OCT) compound and sterile PBS solution was injected to inflate the lung segment to approximately 20 cm/H_2_O of pressure to preserve the tissue architecture during processing. Several serial transverse sections were collected from the inflated segments (less than 0.5 cm thick). The sections were then fixed in 10% neutral buffered formalin and processed in paraffin for histopathological analysis.

Lung samples that were collected for miRNA analysis were flash frozen in liquid Nitrogen and transferred to − 80 °C.

### Histopathological examination

Paraffin-embedded tissue sections (5 μm) were stained with Hematoxylin and Eosin Y (H & E) for general histology and assessment of pathological changes (Fig. 1D). Collagen present in the lung parenchyma was assessed by paraffin sections stained with Masson’s trichrome stain (Trichome stain kit, Abcam (ab150686)) according to manufacturer’s instructions (Fig. 1D).

### Scoring fibrosis

Fibrosis was assessed in the lung tissue sections of sheep according to the scale proposed by Hubner [53]. H & E-stained lung tissue sections were evaluated by capturing, 10 random representative, non-overlapping fields under × 20 magnification. All the images taken from samples for scoring were double-blinded and performed by an experienced veterinary pathologist AS (Fig. 1E).

### Fibrosis fraction

Collagen present in the lung parenchyma was evaluated from the lung tissue sections stained with Masson’s trichrome stain. 10 representative randomly selected, non- overlapping fields without large airways and blood vessels were captured under × 20 magnification. The images were then analyzed using the computer software Image Pro® Plus (Version 6.3.0.512 for Windows, Media Cybernetics, Rockville, Maryland, USA). Color selector tool was used to measure the area of tissue stained with blue (collagen) within the field. The fraction was obtained by dividing the blue color-stained tissue area by the total area of the field and expressed as means and standard errors of mean (Mean ± SEM) (Fig. 1F).

### Statistical analysis

Statistical analysis was performed using GraphPad Prism software, version 8.0.1 for Windows (GraphPad Software, La Jolla California, USA). Lung compliance and the fibrotic fraction were evaluated using Student’s t-test. The degree of fibrosis was analysed using the Mann-Whitney test. The data were expressed as the mean ± standard error of the mean (Mean ± SEM). A *p* value of less than 0.05 (*p* < 0.05) was considered as statistically significant.

### RNA extraction from sheep lung tissues

Five sheep (*n* = 5) with the most severe lung fibrosis were selected based on lung function and lung pathology to evaluate the microRNA expression profiles. Total RNA extraction was performed using the miRNeasy Mini Kit according to manufactures instruction (QIAGEN Pty Ltd., Doncaster, Australia). RNA quality was determined by an Agilent Bioanalyzer 2100.

### microRNA microarray and data analysis

A custom designed ovine miRNA microarray was performed by a service provider LC Sciences, Houston, USA. It contained 21 replicates of 152 unique probes and 55 control probes. The unique probes were derived from identified ovine miRNA sequences downloaded from miRBase (http://www.mirbase.org/) and the microarray included miRNA that have previously been shown to be involved in idiopathic pulmonary fibrosis. Data were background corrected and log2 transformed, with the microarray dataset entered into the GEO database at NCBI (GSE166682) https://www.ncbi.nlm.nih.gov/geo/query/acc.cgi?acc=GSE166682. Statistical analysis was performed for individual sheep separately due to the biological variation. Average intensities from the 21 replicates were taken for each miRNA probe tested in bleomycin and saline/control lung segments. Student’s t-test was performed and *p* < 0.01 was considered statistically significant. Log2 fold change of differentially expressed miRNA in each individual sheep was illustrated using a heatmap (Morpheus software; https://software.broadinstitute.org/morpheus) (Fig. 2). Hierarchical clustering was then performed by applying One minus Pearson correlation to cluster differentially expressed miRNAs. Significant differentially expressed miRNA is shown in Table 1.

### Real-time PCR (qPCR)

qPCR was performed on selected miRNA to validate the miRNA microarray. RNA was isolated using the miRNeasy Mini kit (QIAGEN Pty Ltd., Doncaster, Australia) as mentioned above. miRCURY LNA miRNA PCR starter Kit (QIAGEN Pty Ltd., Doncaster, Australia) was used to perform qPCR according to the manufacturer’s instructions. Primer sequences of the customized miRCURY LNA miRNA PCR assays are listed in Table 4. qPCR parameters for 40 cycles were as follows: initial heat activation 95 °C for 2 min, denaturation 95 °C for 10 s followed by combined annealing 56 °C for 60 s (AriaMx Real- time PCR System). All reactions were performed in a 10 μl reaction volume. miR-103 was used as the housekeeping reference miRNA. Statistical analysis was performed using student’s t-test and *p* < 0.05 was considered statistically significant (Fig. 3).

**Table 4:** Customized primer sequences miRCURY LNA miRNA PCR assay for sheep model of lung fibrosis

microRNA	Primer sequence
miR-30a	5’UGUAAACAUCCUCGACUGGAAGC
miR-21	5’UAGCUUAUCAGACUGAUGUUGAC
miR-29a	5’UAGCACCAUCUGAAAUCGGUU
miR-26a	5’UUCAAGUAAUCCAGGAUAGGCU

### Comparative analysis of miRNA expression profiles of IPF patients, sheep, and mouse models

Journal articles published in English during the period of January 2010 to June 2020 were used for the comparative analysis of the miRNA expression in human and mouse. A systematic search was performed using the search engines “Google Scholar”, “PubMed” and University of Melbourne library catalogue, and the search strategy included the keywords of “microRNA”, “Idiopathic pulmonary fibrosis”, “human” and “mice”. miRNA expression in IPF patients and in vivo expression in the mouse model were considered for this analysis. All the dysregulated miRNA reported in IPF patients, sheep and mouse models were analyzed using the Bioinformatic and Evolutionary Genomics software and presented as Venn diagrams (Fig. 4).

### Gene ontology and KEGG pathway analysis

The biological function of differentially expressed miRNA was determined by Gene Ontology (GO) analysis. The GO terms consist of 3 parts: biological process, cellular component, and molecular function. GO analysis was performed using DIANA mirPath v.3 software [54] by considering the three components mentioned above. DIANA microT-CDS and TargetScan were used to identify miRNA-gene interactions, while the significance was assessed by performing Fisher’s Exact Test. A *p*-value < 0.05 was considered statistically significant (Fig. 5). Kyoto Encyclopedia of Genes and Genomes (KEGG) pathway analysis was also performed using DIANA mirPath v.3 software to evaluate the functions of differentially expressed miRNA and gene interactions. Significance was determined by performing Fisher’s Exact Test. *p* values < 0.05 were considered statistically significant (Fig. 6).

## Data Availability

The microarray data deposited in GEO database at NCBI (GSE166682) https://www.ncbi.nlm.nih.gov/geo/query/acc.cgi?acc=GSE166682.

## Abbreviations

IPF: Idiopathic pulmonary fibrosis
Cseg: Segmental compliance
miRNA: microRNA
GO: Gene Ontology
KEGG: Kyoto Encyclopedia of Genes and Genomes
HPMEC: Human Pulmonary Microvascular Endothelial cells
TGF- β: Transforming growth factor β,
PDGF: Platelet derived growth factor
IL-4-: Interleukin-4
TNF-α: Tumor necrosis factor-α
